# Does livestock protect from malaria or facilitate malaria prevalence? A cross-sectional study in endemic rural areas of Indonesia

**DOI:** 10.1186/s12936-018-2447-6

**Published:** 2018-08-20

**Authors:** Hamzah Hasyim, Meghnath Dhimal, Jan Bauer, Doreen Montag, David A. Groneberg, Ulrich Kuch, Ruth Müller

**Affiliations:** 1 Faculty of Medicine, Institute of Occupational Medicine, Social Medicine and Environmental Medicine, Goethe University, Theodor-Stern-Kai 7, 60590 Frankfurt am Main, Germany; 2 Faculty of Public Health, Sriwijaya University, Indralaya, South Sumatra Indonesia; 3 Nepal Health Research Council, Ramshah Path, Kathmandu, Nepal; 4 Centre for Primary Care and Public Health, Barts and the London School of Medicine, Queen Mary University of London, London, UK

**Keywords:** Malaria, Rural area, Livestock, Zooprophylaxis, Zoopotentation

## Abstract

**Background:**

Ever since it was discovered that zoophilic vectors can transmit malaria, zooprophylaxis has been used to prevent the disease. However, zoopotentiation has also been observed. Thus, the presence of livestock has been widely accepted as an important variable for the prevalence and risk of malaria, but the effectiveness of zooprophylaxis remained subject to debate. This study aims to critically analyse the effects of the presence of livestock on malaria prevalence using a large dataset from Indonesia.

**Methods:**

This study is based on data from the Indonesia Basic Health Research (“Riskesdas”) cross-sectional survey of 2007 organized by the National Institute of Health Research and Development of Indonesia’s Ministry of Health. The subset of data used in the present study included 259,885 research participants who reside in the rural areas of 176 regencies throughout the 15 provinces of Indonesia where the prevalence of malaria is higher than the national average. The variable “existence of livestock” and other independent demographic, social and behavioural variables were tested as potential determinants for malaria prevalence by multivariate logistic regressions.

**Results:**

Raising medium-sized animals in the house was a significant predictor of malaria prevalence (OR = 2.980; 95% CI 2.348–3.782, *P* < 0.001) when compared to keeping such animals outside of the house (OR = 1.713; 95% CI 1.515–1.937, *P* < 0.001). After adjusting for gender, age, access to community health facility, sewage canal condition, use of mosquito nets and insecticide-treated bed nets, the participants who raised medium-sized animals inside their homes were 2.8 times more likely to contract malaria than respondents who did not (adjusted odds ratio = 2.809; 95% CI 2.207–3.575; *P *< 0.001).

**Conclusions:**

The results of this study highlight the importance of livestock for malaria transmission, suggesting that keeping livestock in the house contributes to malaria risk rather than prophylaxis in Indonesia. Livestock-based interventions should therefore play a significant role in the implementation of malaria control programmes, and focus on households with a high proportion of medium-sized animals in rural areas. The implementation of a “One Health” strategy to eliminate malaria in Indonesia by 2030 is strongly recommended.

**Electronic supplementary material:**

The online version of this article (10.1186/s12936-018-2447-6) contains supplementary material, which is available to authorized users.

## Background

Malaria is a life-threatening disease with a widespread and long-term impact on the quality of life and the economy [1, 2]. Infection is caused by the bite of a female *Anopheles* mosquito which is a vector for the *Plasmodium* parasite [3, 4]. In Indonesia, malaria is mostly caused by *Plasmodium vivax* and *Plasmodium falciparum* [5]. Malaria threatens almost half of the world’s inhabitants, around 2.3 billion of which live in Asia [4]. In Indonesia, the national average of malaria prevalence was 2.85% in 2007 and 6.0% in 2013 [6, 7]. Livestock contributes significantly to the livelihoods of hundreds of millions around the world. In Indonesia the percentage of people who keep livestock varies geographically and culturally. Regions of Indonesia where a high percentage of families is involved in raising livestock also had the highest prevalences of clinical malaria in the country (East Nusa Tenggara, 12.0%; Papua, 18.4%) [6].

In the context of malaria, animals can play a role in diverting mosquitoes from feeding on humans, thereby preventing transmission of the parasite to humans [8]. Using alternative host species to distract malaria vectors away from people, a concept known as zooprophylaxis, has long been recommended as a potential environmental strategy to reduce malaria transmission [8]. However, increasing opportunities to feed on alternative hosts such as livestock could also increase human exposure to malaria: an increase in the number of animals living close to mosquito breeding sites, resulting in improved availability of blood meals, could alternatively attract more mosquitoes, increase their survival and the risk of disease transmission to humans, a phenomenon known as zoopotentiation [9]. In such a situation, zooprophylaxis may be ineffective because the effect of diverting blood meal seeking mosquitoes to non-human prey may be countered by higher numbers and longer survival of mosquitoes [8]. Nevertheless, the use of animals as bait to attract mosquitoes has been propagated as a promising alternative to insecticide use. For areas where zoophilic vectors transmit malaria, two types of malaria control approaches using livestock have been suggested; zooprophylaxis and insecticide treatment of livestock (ITL) [10]. As understood in this context, zooprophylaxis is supposed to control vector-borne diseases by withdrawing vectors to livestock species within which the pathogen in question cannot spread. By combining the use of insecticide spray with zooprophylaxis, vector populations in some situations may be controlled without mosquitoes developing insecticide resistance [11]. Increased blood feeding on cattle can reduce the likelihood of human infections in the sense of a zooprophylactic effect [12]. A prophylactic effect of livestock on malaria risk has also been observed in Papua New Guinea and Sri Lanka [10]. In Kenya and Zambia, malaria prevalence became significantly reduced in areas where livestock was kept [9]. Donkeys, rabbits and pigs also showed a significant protective effect [13], possibly because vector breeding sites were closer to livestock enclosures than to houses, and especially endophagic and exophilic *Anopheles* species might prefer to feed on the animals [10]. Accordingly, the presence of cattle could be used as a barrier to the spread of malaria [14, 15]. However, research conducted in Pakistan, the Philippines and Ethiopia showed that the presence of cattle can also be a risk factor for the spread of malaria [10]. The practical value of zooprophylaxis and the reasons for observed zooprophylactic success have therefore remained under debate [10]. Part of the controversy about zooprophylaxis versus zoopotentation for malaria prevalence may be accounted for by the variety of analysed livestock species and animal keeping practices, and the associated variable attractiveness for different zoophilic vectors [9, 10]. For example, zooprophylaxis may more likely take place in areas where livestock is kept at a distance from human sleeping quarters at night, and where nets or other protective measures are used, whereas zoopotentiation may be more likely in places where livestock is housed within or near human sleeping quarters at night and where mosquito species prefer human hosts [16].

The present study addresses the relationship between livestock keeping and malaria prevalence in rural endemic areas of Indonesia. The country has been chosen as the geographical centre for this research because:

1. There is high vector diversity as indicated by the presence of 20 *Anopheles* species [17]. The most abundant malaria vector throughout Indonesia is *Anopheles vagus* (46% at 349 sites), whereas *Anopheles bancroftii* was the geographically most constrained one (1%; 7 locations in Papua, 1 in Maluku) [18].
2. 26.14% of Indonesia’s population live in malaria epidemic environments. Most of the areas at high risk for malaria are rural and located in eastern Indonesia [6].
3. The practice of keeping livestock is widely distributed throughout the Indonesian population. At the national level, 39.4% of households raise poultry, 11.6% raise medium-sized livestock, i.e., goats, sheep, and pigs, 9.0% raise large-sized animals, i.e., cattle, horses, or buffaloes [6], and 12.5% raise other animals such as dogs, cats or rabbits [6].
4. The Indonesian regions where a high proportion of households is involved in raising livestock also presented the highest prevalence of malaria [6]. Abundant livestock can enhance the survival and abundance of mosquitoes, and in this situation zooprophylaxis may become ineffective. Similarly, malaria prevalence was higher among families who kept cattle compared to those who did not [19]. While the larvae of some malaria vectors in Indonesia, such as *Anopheles farauti* sensu lato, were found in a wide variety of temporary man-made and animal-made habitats, such as borrow pits, pig-gardens, and pools along rivers and streams [18], other studies have reported the formation of a barrier between anopheline breeding sites and human residential areas through an active deployment of pigs and cows [19]. However, this example of zooprophylaxis has been discussed in a controversial manner.
  The hypothesis of the present study is that there is indeed a relationship between the presence of livestock and malaria prevalence in rural endemic areas in Indonesia.

## Methods

This study made use of a large dataset based on a cross-sectional survey of the Indonesia Basic Health Research (Indonesia acronym: Riskesdas), in 2007, which is organized by Balitbangkes with a sample framework conducted by the Central Bureau of Statistics (Indonesia acronym: BPS). Riskesdas is a nationwide community-based health research project at the district/city level that is conducted every 5–6 years—a duration that is considered an appropriate interval to assess the development of public health status, risk factors, and the progress of health development efforts.

### Study area

**Fig. 1:**
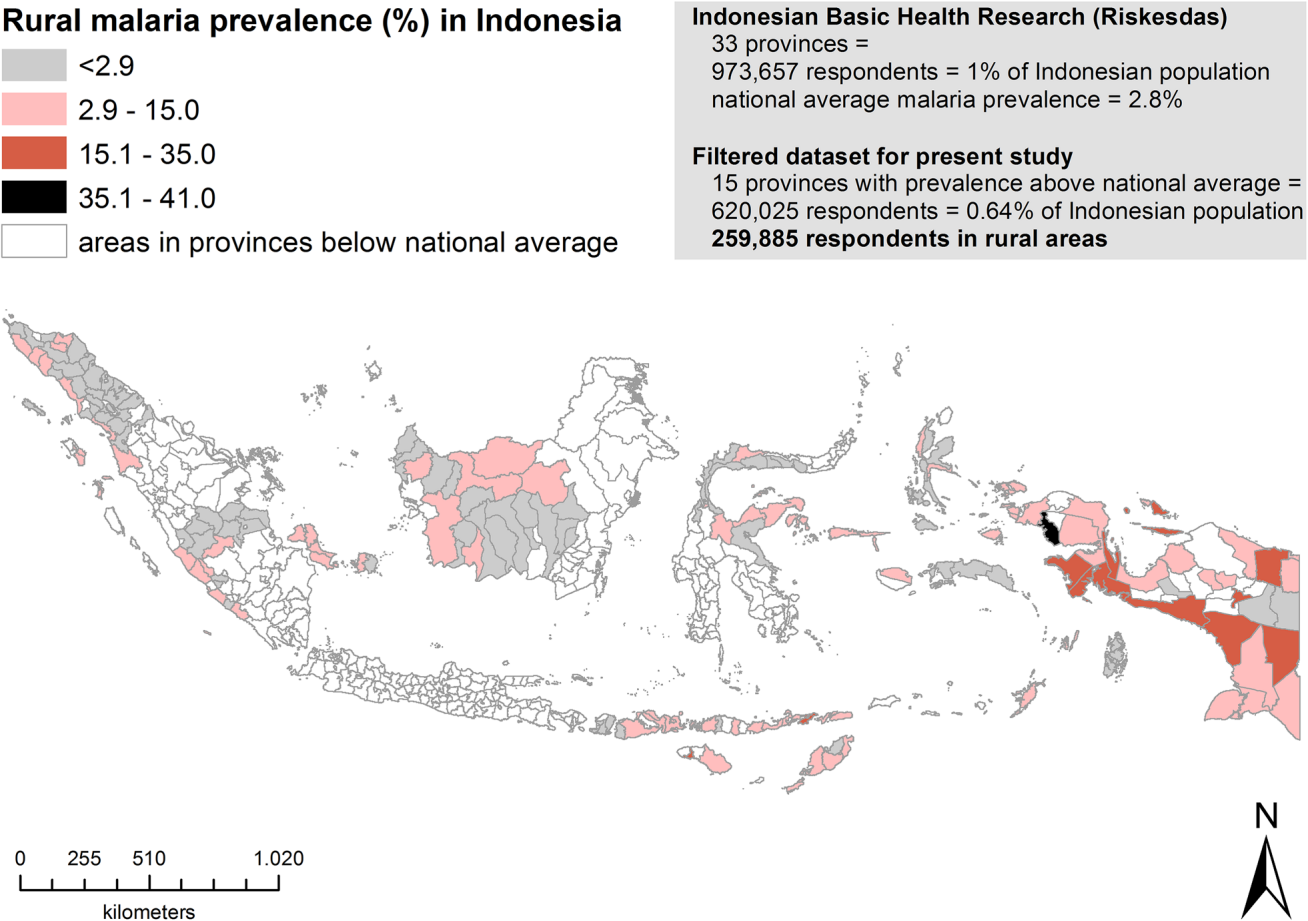
The proportion of malaria in regencies and cities within rural areas of Indonesian provinces with malaria prevalence above the national average

The Riskesdas dataset was filtered for participants residing in the rural areas of 15 highly malaria-endemic (above the national average) provinces (Fig. 1). These 15 provinces include West Papua, Papua, East Nusa Tenggara, Central Sulawesi, North Maluku, Bengkulu, Bangka Belitung, Maluku, West Nusa Tenggara, Nanggroe Aceh Darussalam, Central Kalimantan, West Kalimantan, Jambi, Gorontalo and North Sumatera. Moreover, the provinces of Maluku, North Maluku, West Papua, Papua, and East Nusa Tenggara were highly endemic areas.

### Research variables

The outcome variable, malaria status, is coded as a binary variable whose value equals one if a participant within the past month was ever diagnosed as being malaria-positive by health professionals [6]. Thus the respondent reported having been diagnosed as malaria-positive by a health professional during the past month. In the questionnaire (code B07): in the last 1 month, has [name] ever been diagnosed to suffer from malaria, which was confirmed by a blood test taken by health professionals. Generally, the diagnosis was confirmed by use of rapid diagnostic tests (RDTs) and microscopy in health services. The interviewer did not check for a malaria infection [6]. Further, an independent data collection was taken from an individual and household questionnaire. All the measurements on each person are made at one point in time [20].

The independent variables, such as characteristics of participants (gender, age, education, principal occupation), behaviour of participants (sleep under a mosquito net, use net insecticide, defecating habits), and accessibility and utilization of health services (participants were able to access health services by travelling), environmental sanitation (type of container/media, sewage canal, sewage canal conditions), and location of cages (medium-sized breeding animals and large-sized breeding animals) were tested for a potential relationship with the response variable malaria using the binary category “yes” and “no”. In this study, malaria status include those who have the disease. For a more detailed description of the scope of research variables please refer to Additional file 1.

### Study population

Participants of all ages representative of the entire Republic of Indonesia were interviewed with questions related to malaria. Household samples and household members in Riskesdas 2007 are designed to be identical to households and the household member list in the National Socioeconomic Survey (Indonesia acronym: Susenas) 2007 [6]. Regions designated as rural were used as a survey subsample by the location data retrieval used in the Riskesdas survey 2007 [6]. The analyses in the present research are based on a massive dataset with 259,885 out of 973,657 Riskesdas participants who represent a total population size of 30,152,651 Indonesians.

### Questionnaires

A set of questionnaires was used as an instrument for data collection. The data collection for Riskesdas was done in two stages: the first stage was begun in August 2007 and continued until January 2008 in 28 provinces; the second stage was in August–September 2008 in five provinces (NTT, Maluku, North Maluku, Papua and West Papua). Riskesdas had mobilized 5619 enumerators, all (502) researchers from the National Institute of Health Research, and 86 lectures from technical health schools, local governments in provincial regions and districts/cities, provincial labs, hospitals, and universities were also involved. The process of editing, entry, and cleaning Riskesdas data was started in early January 2008, while there was also a process for discussing work plans and strategies of analysis. Various questions related to Indonesian health policy were research questions and were finally developed to become variables collected by using several approaches. In Riskesdas 2007, there are around 900 variables spread out in six kinds of questionnaires. The questionnaires covered malaria and included 14 explanatory variables. Regarding raising livestock, data were collected by asking all heads of households whether they were keeping poultry, medium-sized livestock (goats, sheep, and pigs), large-sized livestock (cows, buffaloes, and horses) or pets such as dogs, cats, and rabbits. If livestock was kept, then it was noted whether the livestock was kept inside of the house or outdoors [6].

### Statistical analyses

Data were analysed using statistical data processing applications by Stata, taking into account the complex sampling design (using two-stage sampling, for a more detailed description of statistical procedure please refer to Additional file 1). By using a Stata complex sample in processing and analysing Riskesdas data, the validity of analysis result can be optimized. Both univariate and bivariate analyses were carried out using Chi square tests. In the next stage of multivariable analysis, a series of binary logistic regressions were run. Explanatory variables that may have predictive value for the response variable were selected for the multiple regression models (Wald test, *P *< 0.25) [21].

Analysis of multivariable logistic regression was carried out to specify the relationship amongst multiple independent variables with the dependent variable ‘malaria prevalence’. The final model includes the following seven explanatory variables: characteristics of participants (gender, age), community health facility, the condition of sewage canal, the behaviour of participants (using mosquito nets, and insecticide-treated mosquito nets), and raising medium-sized breeding animals). In Table 2, the adjusted odds ratio (AOR), as a result of parsimonious logistic models, is shown for independent variables affecting the prevalence of malaria in rural endemic areas of 15 high malaria-endemic provinces of Indonesia.

## Results

### Malaria prevalence

Prevalence of malaria in Indonesia in 2007, shown in Fig. 1, revealed that malaria prevalence was 3.5% (95% CI 0.033–0.037) in 15 provinces with malaria prevalence higher than the national average (2.85% in 2007) [6]. The study area map uses the World Geodetic System (WGS84) as its reference coordinate system. The mapping of malaria prevalence based on Riskesdas data was performed using the software Aeronautical Reconnaissance Coverage Geographic Information System (ArcGIS 10). The highest malaria prevalence found was 41.0% at South Sorong (marked as a black area in Fig. 1), a regency located in the West Papua province of Indonesia with an area of 3946.94 km^2^ and a population of 37,900 (2010 census).

### The existence of livestock

**Fig. 2:**
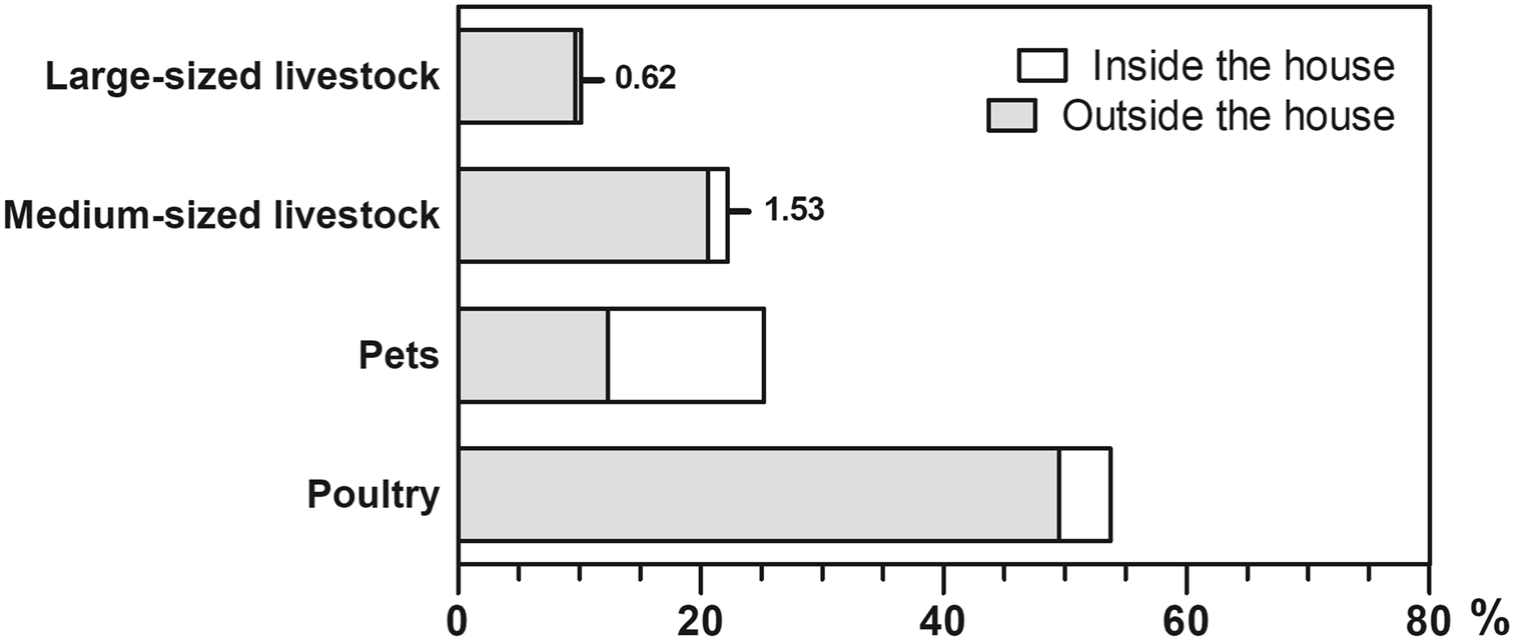
The proportion of rural population (n = 259,885 household members) raising livestock (%) and the location of cages (inside the house—white bar, outside the house—grey bar) in highly malaria-endemic endemic areas in 15 provinces of Indonesia. The category of poultry includes chicken, ducks and birds. The category of pets includes dogs, cats and rabbits. The category of medium-sized breeding animals includes goats, sheep and pigs. The category of large-sized breeding animals includes cows, buffaloes and horses

Based on the Riskesdas questionnaire, the animals are categorized as livestock, pets and poultry. The term livestock includes large-sized breeding animals (cattle, horses, buffaloes), and medium-sized breeding animals (goats, sheep, pigs). Additionally, poultry, such as chicken and ducks, and pets, such as dogs, cats and rabbits, are included in the term *pets*. With 53.7%, the majority of participants raises chickens, ducks, and birds, followed by pets (dogs, cats, and rabbits; 25.2%), medium-sized breeding animals (goats, sheep, and pigs; 22.2%), and large-sized breeding animals (cows, buffaloes, and horses; 10.2%) (Fig. 2). This research further analysed the raising of both large-sized breeding animals (cattle, horses, buffaloes) and medium-sized breeding animals (goats, sheep, pigs) that are connected with malaria prevalence. This research inevitably reveals that 0.52% (95% CI 0.004–0.007) of participants keep large-sized breeding animals and 1.63% (95% CI 0.014–0.019) of participants keep medium-sized breeding animals inside the house. This study also found that 9.64% (95% CI 0.091–0.102) of the participants keep large-sized breeding animals, and 20.59% (95% CI 0.197–0.215) participants keep medium-sized breeding animals outside of the house. Livestock kept in close proximity to humans can contribute to the higher transmission, as they attract mosquitoes into areas where they will encounter and feed on human hosts opportunistically (zoopotentiation) [22].

### Univariate and bivariate analysis

Table 1 summarizes the percentage of participants having or not having been diagnosed positive for malaria for each of the explanatory variables and bivariate analyses (for more details see Additional file 2). In brief, this survey observes the participants who keep large-sized breeding animals inside of the house (0.52%, 95% CI 0.004–0.007), and the participants who keep the animals outside of the house (9.64%, 95% CI 0.091–0.102). It additionally observes, participants who keep medium-sized breeding animals inside of the house (1.63%, 95% CI 0.014–0.019), and the participants who keep the animals outside the house (20.59%, 95% CI 0.197–0.215). Furthermore, Table 2 shows that malaria prevalence is increased in the participants who keep medium-sized breeding animals inside of the house (OR = 2.980; 95% CI 2.348–3.782, *P* < 0.001), and the participants who keep the animals outside of the house (OR = 1.713; 95% CI 1.515–1.937, *P* < 0.001) and who contract malaria more than those who do not have such animals. On the contrary, keeping large-sized breeding animals does not considerably increase malaria prevalence. Besides, males are more likely to have malaria than females (OR = 0.849, 95% CI 0.811–0.888, *P* < 0.001). Participants who are aged 15–64 years (OR = 0.861, 95% CI 0.812–0.912, *P* < 0.001) contract malaria more than those who have not yet reached that age. In addition, most participants who were able to access health services by travelling for more than 60 min (OR = 1.633, 95% CI 1.251–2.131, *P* < 0.001) were more susceptible to contract malaria than participants who were able to access health services by travelling less than 60 min. The majority of participants who use open sewage systems (domestic wastewater or municipal wastewater) at home and those without a sewage system are at higher odds of contracting the disease (OR = 1.250, 95% CI 1.095–1.427, *P* = 0.001) than participants who have closed sewage systems. Participants who were using mosquito nets with OR = 0.805 and insecticide-treated bed nets (ITNs) with OR = 0.508 as protective factors against malaria reveal a decreased malaria prevalence compared to those who do not use such protection. Besides, there was a negative association between the use of insecticide-treated bed nets and the prevalence of malaria (r = − 0.023, *P* < 0.001). This statistic implies for participants who increasingly used ITNs that the prevalence of malaria decreased.

**Table 1:** Description of variables research (%) within the categorical variable: malaria prevalence, characteristics of participants, the accessibility and utilization of health service, environmental sanitation, the behaviour of participants, and the location of cages of livestock

Variable research with n = 259,885	Proportion (%)	95% CI	
		Lower	Upper
The *dependent* variable			
Malaria prevalence			
0. No	96.53	0.963	0.967
1. Yes	3.47	0.033	0.037
The *independent* variables			
Sex			
0. Male	49.29	0.491	0.495
1. Female	50.71	0.505	0.509
Age (years)			
0. Productive age (15–64 years)	60.09	0.598	0.604
1. Not productive age (< 15 and > 64 years)	39.91	0.396	0.402
Education			
0. Completed high school	12.42	0.12	0.128
1. High school not completed	63.98	0.636	0.644
2. < 10 years of age	23.60	0.234	0.238
Main occupation			
0. Other occupation	45.43	0.449	0.46
1. Farmer/fisherman/labourer	30.97	0.304	0.315
2. < 10 years of age	23.60	0.234	0.238
The time to reach the nearest hospital			
0. < 60 min	93.18	0.925	0.938
1. > 60 min	6.82	0.062	0.075
The time to reach the nearest community health facilities			
0. < 60 min	95.24	0.947	0.957
1. > 60 min	4.76	0.043	0.053
The type of container/media used			
0. Closed container	62.57	0.614	0.637
1. Others	37.43	0.363	0.386
The sewage canal			
0. Closed container in the yard	5.52	0.051	0.06
1. Others	94.48	0.94	0.949
The condition of sewage canal			
0. Closed canal	9.92	0.094	0.105
1. Others	90.08	0.895	0.906
Mosquito nets			
0. Yes	43.99	0.428	0.452
1. No	55.22	0.54	0.564
2. No answer	0.79	0.007	0.009
Insecticide-treated bed net			
0. Yes	11.43	0.107	0.122
1. No	29.01	0.279	0.301
2. No answer	59.56	0.584	0.607
The habit of defecate			
0. Yes	44.29	0.433	0.453
1. No	32.11	0.312	0.33
2. < 10 years of age	23.60	0.234	0.238
Raising large-sized breeding animals (cows, buffaloes, horses)			
0. No have	89.84	0.892	0.904
1. Cage inside the house	0.52	0.004	0.007
2. Cage outside the house	9.64	0.091	0.102
Raising medium-sized breeding animals (goats, sheep, pigs)			
0. No have	77.78	0.768	0.788
1. Cage inside the house	1.63	0.014	0.019
2. Cage outside the house	20.59	0.197	0.215

**Table 2:** The logistic regression analysis associated with the prevalence of malaria in rural highly malaria-endemic endemic areas in 15 provinces of Indonesia, with n = 259,885

Risk factor	*P*-value	Unadjusted	*P*-value	Adjusted
		OR (95% CI)		OR (95% CI)
Sex				
Male versus female	0.000	0.849 (0.811–0.888)	0.000	0.842 (0.804–0.882)
Age (years)				
Productive age (15–64 years) versus not productive age (< 15 and > 64 years)	0.000	0.861 (0.812–0.912)	0.000	0.837 (0.790–0.887)
Community health facility				
< 60 min versus > 60 min	*0.000*	*1.633 (1.251*–*2.131)*	*0.005*	*1.446 (1.120*–*1.866)*
The condition of sewage canal				
Close canal versus others	*0.001*	*1.250 (1.095*–*1.427)*	*0.015*	*1.177 (1.033*–*1.343)*
Mosquito nets				
Yes versus not	0.000	0.805 (0.727–0.890)	0.157*	0.879 (0.736–1.051)
Yes versus others	*0.002*	*1.911 (1.273*–*2.868)*	*0.005*	*1.838 (1.208*–*2.797)*
Insecticide-treated bed net				
Yes versus not	0.000	0.508 (0.439–0.588)	0.000	0.509 (0.440–0.589)
Yes versus others	0.000	0.527 (0.457–0.608)	0.000	0.590 (0.481–0.725)
Raising medium-sized breeding animals				
Not have versus inside	*0.000*	*2.980 (2.348*–*3.782)*	*0.000*	*2.809 (2.207*–*3.575)*
Not have versus outside	*0.000*	*1.713 (1.515*–*1.937)*	*0.000*	*1.643 (1.460*–*1.849)*

Risk factors with *P* < 0.001 or *P* < 0.05 and OR > 1 are shown in italic face

*P > 0.05 a confounding factor

### Multivariable logistic regression

The estimated AOR of malaria for participants who kept medium-sized breeding animals (goats, sheep, pigs) inside at home signifies a 2.81 times higher risk of contracting malaria (adjusted for other variables; AOR = 2.809; 95% CI 2.207–3.575; *P* < 0.001) in rural endemic areas of 15 highly malaria-endemic provinces of Indonesia. The other six controlling factors for malaria prevalence relate to sociodemographic factors, socioeconomics and behaviour.

## Discussion

In the present study, the presence of medium-sized livestock increased the likelihood of contracting malaria by 2.81. The results of this study therefore suggest that the presence of certain livestock types potentiate malaria risk. Other principal factors affecting the prevalence of malaria were demographic factors such as gender, age, access to health facility, environmental health, and the behaviour of participants concerning protection against malaria by means of mosquito nets and ITNs.

### Spatial heterogeneity of malaria prevalence

Spatial variation in malaria prevalence has to be taken into account in Indonesia [23]. The highest malaria prevalence was found in South Sorong, a known malaria endemic province [6]. A gradient of malaria prevalence from rural (58.9%) to urban areas (33.9%) has been known in the Bata district of Equatorial Guinea (EG) [24]. This situation is consistent with the identified high-risk in the rural context that was found in West Papua, Papua [23] and East Nusa Tenggara [6, 25]. A similar variation of spatial malaria distribution was observed in a cross-sectional study in rural areas in Haiti (4–41%), and demographic data indicated some focal disease transmission [26].

### Keeping medium-sized animals is a significant determinant for malaria prevalence

This investigation provides evidence for a positive relationship between medium-sized animals that are kept inside the house (AOR = 2.809; 95% CI 2.207–3.575; *P* < 0.001) and the prevalence of malaria in the human population living in rural, highly malaria endemic areas of Indonesia. An explanation for these results could be that the presence of livestock increased the abundance of vectors for *Plasmodium* species. Increasing the availability of host selection for certain livestock could increase human malaria exposure by means of zoopotentiation if the heat and odour cues emitted by animals attract a higher number of vectors to households in or near the area where they are kept [9]. Zoopotentiation could also occur if the physical disturbances created by animals (e.g., puddles, hoof prints, watering sites) increase the potential for larval habitats and thus adult vector density near households. In this study, the participants who had an open sewage canal were at higher odds of contracting malaria than others, highlighting the importance of potential larval habitats near houses. The splitting of people and livestock dwellings on this scale proves to be too large to dodge a zoopotentiation effect [9]. An increasing abundance of goats or sheep has been demonstrated to increase the abundance of *Anopheles* mosquitoes within a radius of 20 m around the household in Kenya [12]. Other evidence for zoopotentation includes positive correlations between donkeys, pigs, and humans, and the abundance of malaria-transmitting mosquitoes [12, 27]. For example, the probability that humans are bitten by the zoophilic *Anopheles stephensi* may increase if one sleeps close to a cow or a goat in the evenings. In contrast, the anthropophily of *Anopheles culicifacies* was only slightly influenced by the presence of livestock. In Kenya, each additional goat or sheep increased the abundance of the local malaria vector [12], and one may assume that there was a higher human biting rate as well. At least participants who kept pigs and sheep in Mozambique had significantly increased odds of malaria infection, although to a lesser extent in the case of sheep [27]. For the zoophilic *An. stephensi*, nightly human biting increased by 38% in the presence of a cow and by 50% in the presence of two goats [19]. An integrative vector control strategy including ITNs and indoor residual spraying (IRS) reduction, combined with ITL, may improve zooprophylactic effectiveness [28].

### Keep livestock at a distance

In particular, participants who were raising medium-sized breeding animals inside their home were more likely to have malaria (OR = 2.980; 95% CI 2.348–3.782; *P* < 0.001), and participants who were raising medium-sized breeding animals outside their home were more likely to have malaria (OR = 1.713; 95% CI 1.515–1.937; *P* < 0.001) than those who did not raise the livestock. In contrast to the outcome of the study, livestock may indeed have a prophylactic effect in cases in which only zoophilic vectors are present and livestock is placed in a way to act as a protective barrier for anopheline mosquitoes [10]. Otherwise, zoopotentation often takes place when livestock is kept indoors or near the household and if mosquito vectors are mainly anthropophilic [16]. A parallel approach of insecticide-treated livestock (ITL) and arranging the livestock as far from man as possible is sufficient to reduce malaria [10, 19]. Likewise, in the Macha area in the southern province of Zambia, farm animals revealed a dramatically declining risk of *P. falciparum* infection at the house level, with an increasing distance between livestock (cattle, goats, dogs, cats) and dwelling structures.

### Demographic and social determinants of malaria status

Participants in the age range of 15–64 years, and especially male participants, contracted malaria significantly more than others. Malaria prevalence also differs by gender, with men more likely to be parasitaemic than older women in the Democratic Republic of Congo [29]. Similarly, in a larger scaled survey of households in Ethiopia, the frequency of suspected malaria in men was significantly higher than in women; however, the prevalence of malaria was not significant between genders [30]. In contrast, women in the adult population of an endemic area in Kenya are 50% more likely to become infected with malaria parasites than men [31].

### Behavioural determinants of malaria status

Protective behaviour (mosquito nets and ITNs) can reduce the risk of malaria. In rural, highly malaria endemic areas of Indonesia, the risk of contracting malaria significantly decreased if ITNs were used. Similarly, ITNs are the most protruding prevention of malaria in highly endemic areas in Malaysia [32], along with other community-based preventive measures, such as bed nets [33]. Furthermore, ITNs and long-lasting insecticidal nets (LLINs) were combined with indoor residual spraying to accelerate success in malaria control in tropical Africa [34]. Seemingly using of ITNs in 2007 is not more effective for as protection for malaria with (r = − 0.023, *P* < 0.001), due to the number of ITNs distributed at the time, the number of people protected is low, and lack of good behaviour of the community regarding the use of ITNs in the research area [17, 35]. Furthermore, the malaria program has been using long-lasting insecticidal nets (LLIN), which are more effective than ITNs. LLINs have been used significantly more as an effective alternative to ITNs for over a decade [36].

## Limitations of research

A weakness of our study is that the clinical diagnosis of malaria by retrospective interview of last 4 weeks may underestimate malaria positive respondents. We expect that if we would increase the period for clinical diagnosis, more people would report positive malaria diagnosis. The cross-sectional design cannot decide how the chances of getting malaria for participants were before and after exposure to covariate variables. However, the benefits of a large-scale cross-sectional design are the increase in information on preliminary phenomena which subsequently allows for designing studies with particular foci [37]. There are other factors also proven to determine malaria prevalence, such as the bionomics of different *Anopheles* species [38]. Understanding the kind of *Anopheles* species, and the behaviour of *Anopheles* mosquitoes can help conceive how malaria is transmitted and can assist in designing appropriate control strategies. Unfortunately, in the Riskesdas 2007, these factors were not monitored.

### Recommendations

In this study, participants who raised medium-sized animals inside their homes had a higher malaria prevalence in 15 provinces throughout the rural malaria endemic areas of Indonesia. Hence, the main recommendation from this study is to keep this livestock outside of the house, and to focus livestock-based interventions on households with a high proportion of medium-sized animals in rural malaria endemic areas of Indonesia. In this context, anthropological studies should be undertaken to understand in the first place why people in different parts of Indonesia are keeping livestock the way they do. Participatory community eco-health approaches might be best suited to work with local people and communities in order to develop a lasting intervention together, since a vertical policy might not be successful [39–41].

Besides, participants aged 15–64 years should be provided with the means for protection from *Anopheles* bites while working in rural malaria endemic areas, including personal protection, behaviour modification and environmental modification. Personal protection includes using insecticides and repellent and the use of long-sleeved clothing and trousers. Environmental modification is aimed at reducing mosquito habitats, covering leaky rooves, among others. There is also a need for improving sanitation by closing sewage canals to reduce the breeding places of *Anopheles* mosquitoes. Seemingly using of ITNs in 2007 is not more effective for as protection for malaria with (r = − 0.023, *P* < 0.001). This study therefore recommends the distribution of LLINs to all people in rural endemic areas together with community-based interventions to improve the knowledge, attitude and practical use and maintenance of LLINs for malaria prevention.

## Conclusions

The presence of medium-sized livestock (goats, sheep, and pigs), is the major risk factor for contracting malaria in rural malaria endemic areas of Indonesia. Sociodemographic and behavioural factors are also important for having a high risk of malaria infection. Thus, livestock-based interventions should be prioritized in Indonesia and focus on households with a high proportion of medium-sized animals in malaria endemic rural areas. ‘One Health’ community research approaches that encompass the understanding of local perceptions of malaria, malaria transmission and livestock as well as the use of preventive tools like long-lasting insecticide impregnated bed nets should be strengthened in Indonesia to inform the adequate development of an integrative malaria prevention strategy.

## Additional files

**Additional file 1.** Detailed description of scope of variables and statistical procedure.

**Additional file 2.** Detailed description of descriptive analysis and bivariate analysis.

## Abbreviations

AOR: adjusted odds ratio
API: annual parasite incidence
ArcGIS: Aeronautical Reconnaissance Coverage Geographic Information System
IDHS: Indonesian Demographic and Health Survey
IRS: indoor residual spraying reduction
ITL: insecticide-treated livestock
ITNs: insecticide-treated mosquito nets
IVM: Integrated vector management
LLINs: long-lasting insecticidal net
MHD: man-hour density
MOH: Ministry of Health
NIHRD: National Institute of Health Research and Development
NTT: East Nusa Tenggara
OR: odds ratio
PHCs: Primary health centres
RDTs: rapid diagnostic tests
Riskesdas: Indonesia basic health research (Indonesia acronym: Riset kesehatan Dasar)
Ristekdikti: Ministry of Research, Technology and Higher Education (Indonesia acronym: Ristekdikti)
Susenas: National Socioeconomic Survey (Indonesia acronym: Susenas)
USAID: US Agency for International Development
VBDs: vector-borne diseases
WGS84: World Geodetic System 1984

## References

[1] Schwake L, Streit JP, Edler L, Encke J, Stremmel W, Junghanss T (2008). Early treatment of imported falciparum malaria in the intermediate and intensive care unit setting: an 8-year single-center retrospective study. Crit Care.

[2] Tambo E, Adedeji AA, Huang F, Chen J-H, Zhou S-S, Tang L-H (2012). Scaling up impact of malaria control programmes: a tale of events in Sub-Saharan Africa and People’s Republic of China. Infect Dis Poverty.

[3] Ministry of Health Republic of Indonesia (2014). Malaria management: guideline.

[4] Tanner M, Greenwood B, Whitty CJ, Ansah EK, Price RN, Dondorp AM (2015). Malaria eradication and elimination: views on how to translate a vision into reality. BMC Med.

[5] Elyazar IR, Gething PW, Patil AP, Rogayah H, Sariwati E, Palupi NW (2012). *Plasmodium vivax* malaria endemicity in Indonesia in 2010. PLoS ONE.

[6] National Institute of Health Research and Development (2008). Indonesia Basic Health Research (RISKESDAS) 2007.

[7] National Institute of Health Research and Development (NIHRD) (2014). Indonesia Basic Health Research (RISKESDAS) 2013.

[8] Saul A (2003). Zooprophylaxis or zoopotentiation: the outcome of introducing animals on vector transmission is highly dependent on the mosquito mortality while searching. Malar J.

[9] Mayagaya VS, Nkwengulila G, Lyimo IN, Kihonda J, Mtambala H, Ngonyani H (2015). The impact of livestock on the abundance, resting behaviour and sporozoite rate of malaria vectors in southern Tanzania. Malar J.

[10] Franco AO, Gomes MG, Rowland M, Coleman PG, Davies CR (2014). Controlling malaria using livestock-based interventions: a one health approach. PLoS ONE.

[11] Kawaguchi I, Sasaki A, Mogi M (2004). Combining zooprophylaxis and insecticide spraying: a malaria-control strategy limiting the development of insecticide resistance in vector mosquitoes. Proc Biol Sci.

[12] Iwashita H, Dida GO, Sonye GO, Sunahara T, Futami K, Njenga SM (2014). Push by a net, pull by a cow: can zooprophylaxis enhance the impact of insecticide treated bed nets on malaria control?. Parasit Vectors.

[13] Bulterys PL, Mharakurwa S, Thuma PE (2009). Cattle, other domestic animal ownership, and distance between dwelling structures are associated with reduced risk of recurrent *Plasmodium falciparum* infection in Southern Zambia. Trop Med Int Health.

[14] Do Manh C, Beebe NW, Van Thi VN, Le Quang T, Lein CT, Van Nguyen D (2010). Vectors and malaria transmission in deforested, rural communities in North-Central Vietnam. Malar J.

[15] Murhandarwati EEH, Fuad A, Nugraheni MD, Wijayanti MA, Widartono BS, Chuang T-W (2014). Early malaria resurgence in pre-elimination areas in Kokap Subdistrict, Kulon Progo, Indonesia. Malar J.

[16] Donnelly B, Berrang-Ford L, Ross NA, Michel P (2015). A systematic, realist review of zooprophylaxis for malaria control. Malar J.

[17] Elyazar IR, Hay SI, Baird JK (2011). Malaria distribution, prevalence, drug resistance and control in Indonesia. Adv Parasitol.

[18] Elyazar IR, Sinka ME, Gething PW, Tarmidzi SN, Surya A, Kusriastuti R (2013). The distribution and bionomics of anopheles malaria vector mosquitoes in Indonesia. Adv Parasitol.

[19] Hewitt S, Kamal M, Muhammad N, Rowland M (1994). An entomological investigation of the likely impact of cattle ownership on malaria in an Afghan refugee camp in the North West Frontier Province of Pakistan. Med Vet Entomol.

[20] Mann C (2003). Observational research methods. Research design II: cohort, cross sectional, and case–control studies. Emerg Med J.

[21] Bursac Z, Gauss CH, Williams DK, Hosmer DW (2008). Purposeful selection of variables in logistic regression. Source Code Biol Med.

[22] Waite JL, Swain S, Lynch PA, Sharma SK, Haque MA, Montgomery J (2017). Increasing the potential for malaria elimination by targeting zoophilic vectors. Sci Rep.

[23] Hanandita W, Tampubolon G (2016). Geography and social distribution of malaria in Indonesian Papua: a cross-sectional study. Int J Health Geogr.

[24] Ncogo P, Herrador Z, Romay-Barja M, Garcia-Carrasco E, Nseng G, Berzosa P (2015). Malaria prevalence in Bata district, Equatorial Guinea: a cross-sectional study. Malar J.

[25] Mulyono A, Alfiah S, Sulistyorini E, Negari KS (2013). Hubungan keberadaan ternak dan lokasi pemeliharaan ternak terhadap kasus malaria di Provinsi NTT (analisis lanjut data Riskesdas 2007). Vektora Jurnal Vektor dan Reservoir Penyakit.

[26] Elbadry MA, Al-Khedery B, Tagliamonte MS, Yowell CA, Raccurt CP, Existe A (2015). High prevalence of asymptomatic malaria infections: a cross-sectional study in rural areas in six departments in Haiti. Malar J.

[27] Temu EA, Coleman M, Abilio AP, Kleinschmidt I (2012). High prevalence of malaria in Zambezia, Mozambique: the protective effect of IRS versus increased risks due to pig-keeping and house construction. PLoS ONE.

[28] Asale A, Duchateau L, Devleesschauwer B, Huisman G, Yewhalaw D (2017). Zooprophylaxis as a control strategy for malaria caused by the vector *Anopheles arabiensis* (Diptera: Culicidae): a systematic review. Infect Dis Poverty.

[29] Messina JP, Taylor SM, Meshnick SR, Linke AM, Tshefu AK, Atua B (2011). Population, behavioural and environmental drivers of malaria prevalence in the Democratic Republic of Congo. Malar J.

[30] Yimer F, Animut A, Erko B, Mamo H (2015). Past five-year trend, current prevalence and household knowledge, attitude and practice of malaria in Abeshge, South-Central Ethiopia. Malar J.

[31] Jenkins R, Omollo R, Ongecha M, Sifuna P, Othieno C, Ongeri L (2015). Prevalence of malaria parasites in adults and its determinants in malaria endemic area of Kisumu County, Kenya. Malar J.

[32] Killeen GF, Smith TA, Ferguson HM, Mshinda H, Abdulla S, Lengeler C (2007). Preventing childhood malaria in Africa by protecting adults from mosquitoes with insecticide-treated nets. PLoS Med.

[33] Yamamoto SS, Louis VR, Sie A, Sauerborn R (2009). The effects of zooprophylaxis and other mosquito control measures against malaria in Nouna, Burkina Faso. Malar J.

[34] World Health Organization (2013). Malaria entomology and vector control. Guide for participants.

[35] Statistics Indonesia (Badan Pusat Statistik—BPS) and Macro International (2008). Indonesia Demographic and Health Survey 2007.

[36] G-G Yang, Kim D, Pham A, Paul CJ (2018). A Meta-regression analysis of the effectiveness of mosquito nets for malaria control: the value of long-lasting insecticide nets. Int J Environ Res Public Health.

[37] Sedgwick P (2014). Ecological studies: advantages and disadvantages. BMJ.

[38] Lowe R, Chirombo J, Tompkins AM (2013). Relative importance of climatic, geographic and socio-economic determinants of malaria in Malawi. Malar J.

[39] Charron DF (2012). Ecohealth research in practice.

[40] Charron DF (2012). Ecosystem approaches to health for a global sustainability agenda. EcoHealth.

[41] Mitchell-Foster K, Ayala EB, Breilh J, Spiegel J, Wilches AA, Leon TO (2015). Integrating participatory community mobilization processes to improve dengue prevention: an eco-bio-social scaling up of local success in Machala, Ecuador. Trans R Soc Trop Med Hyg.

