# Supplementary material for: Does livestock protect from malaria or facilitate malaria prevalence? A cross-sectional study in endemic rural areas of Indonesia
Source: Malar J. 2018 Aug 20;17:302. doi: 10.1186/s12936-018-2447-6 (PMC6102806; doi:10.1186/s12936-018-2447-6)
Supplement: Supplementary file 1 — Additional file 1. Detailed description of scope of variables and statistical procedure. [file 12936_2018_2447_MOESM1_ESM.docx]

## Appendix S1: Detailed description of scope of variables and statistical procedure.

## Scope of variables

*Data management.* Data were managed with Stata software as following: For the dependent variable (malaria), code = 0 indicated healthy participants [no malaria] and code = 1 indicated [have malaria]. Likewise for the independent variables, a “small” code was given to describe a variable as “good condition” or a “group that is not at risk”. Reference was set to code = 0. Automatically, Stata treated the lowest code in the comparison group as a reference category.

*Malaria prevalence.* In this study, "having malaria" was defined as participants who have ever been diagnosed with malaria by health workers. Malaria infection was not tested during the interview; this way unrecorded malaria cases cannot be excluded. Malaria was placed into categories, "malaria" or "no malaria" as binary logistics. The data were obtained from the retrospective assessment by health surveyors using a standardised questionnaire.

*Characteristics of participants.* Gender differences were divided into male and female and were taken from the questionnaire *RKD07. RT block IV no.4*. Age was the lifespan of the participants in years; if age was <1 years "00" was filled in and if the age was ≥97 years "97" was filled in based on the age of the population, according to demographic characteristics. Age categories were created, defined as productive (15-64 years) and not productive (<15 and >64 years). Education was defined as the highest level of education attained by participants. Upon completion of high school education, participants were considered as higher educated and coded = 0. However, participants who had not completed high school education were seen as low educated and given a code = 1 and if the respondent <10 years were given the code = 2. These data were taken from questionnaire *RKD RT block IV no.7*. The main occupation of participants was taken from questionnaire *RKD07. RT block IV no.8,* and we divided this variable into three categories. If the main employment of a respondent was not a farmer /fisherman/ labour, then this was given a code = 0. If the main employment was as a farmer /fisherman/ labour, this was given a code = 1, and if the respondent was <10 years, the code = 2 was given.

*The accessibility & utilisation of health service.* From questionnaire *b6r1b*, time to the hospital was given a code = 0 if the respondent had good access, i.e. if the travel time = < 60 minutes and coded = 1 if travel time was > 60 minutes. The same categories for coding were used for data from questionnaire *b6r2b* to categorise time to the primary health care.

*Environmental sanitation.* According to questionnaire *d09*, the variable of defecating was given a code = 0 if participants were in the habit of defecating in toilets and a code = 1 if participants did not use toilets or gave no answer. Environmental sanitation, such as the type of container/reservoir used, was given a code = 0 if the container/reservoir was closed or given a code = 1 if not. From questionnaire *b7r9*, the variable of a sewage canal was given a code = 0 if the sewage canal was closed or given a code = 1 if not. In questionnaire *b7r10*, the variable of a chemical sewage canal was given a code = 0 if there was good sanitation (sewerage closed) or given a code = 1 if not.

*Behaviour of participants.* From questionnaire *b4k10*, the use of mosquito nets was categorised as follows: If participants used mosquito nets at night, these were given a code = 0. If participants did not use mosquito nets, then these were given a code = 1, while if the respondent gave no answer, these were given a code = 2. From questionnaire *b4k11*, the variable of insecticide-treated nets (ITNs) was investigated. Those participants using ITNs were given a code = 0, while if ITNs were not used, these were given a code = 1, and if the participants did not answer or did not sleep using mosquito nets, these were given a code = 2.

*The existence of livestock and location of cages.* These independent variables were taken from questionnaire *VII.16 at number 16 (1)* and *VII.16 at number 16 (2) b and c* and involved those participants who raised poultry including chicken, ducks and birds, pets including dogs, cats and rabbits and who kept livestock, raising both medium sized breeding animals (goats, sheep, pigs, etc.) and large sized breeding animals (cows, buffaloes, horses, etc.). The location of livestock sheds where participants kept breeding animals and was categorized into “cage in the house“, “cage outside the house“, “household without indoor cage“ and “houses that kept animals outdoors without a cage“, or the respondent did not have cattle. Variables with >2 groups were transformed into dummy variables.

## Details of data analysis

Data were analysed using statistical data processing applications by Stata taking into account the complex sampling design ([Hosmer Jr & Lemeshow, 2004](#_ENREF_26)). Data about the proportion of participants with malaria prevalence, characteristics of participants, their accessibility and utilisation of health service, environmental sanitation, the behaviour of participants, and the existence of livestock/pets and location of cages were analysed. In bivariate analysis, we use two-way tables for survey data. Survey estimation commands are governed by the svy prefix. The svy option is used with many statistical commands to adjust for the effect of sample design when analysing survey data. The *svy: tabulate* uses *-tabdisp- display tables* command to produce the table. The main difference is that *svy: tabulate* computes a test of independence that is appropriate for complex survey data (Minot N. 2009). The *svyset* manages for survey analysis settings of a dataset to designate variables that contain information about the survey design, such as the sampling units and weights. (Stata Corp LP. 2017). By running a series of bivariate logistic regressions, independent variables that may have predictive value for the dependent variable were selected for the multiple regression model (model 1) (Wald test, P < 0.25) ([Bursac et al., 2008](#_ENREF_12)). Also, the statistically insignificant variable "raising of large sized breeding animals" was included in the multiple regression model (model 1) ([Archer & Lemeshow, 2006](#_ENREF_4)). To determine the relationship amongst multiple independent variables with the dependent variable malaria prevalence, a final multiple logistic regression analysis (model 2) was computed with the significant explanatory factors from the multiple regression model 1. Confounder variables with a >10% change of odd ratio and p < 0.05 were identified: The variables “raising of large sized breeding animals” and “location of cages”, either indoors or outdoors, possessed co-linearity and hence were omitted from the final regression analysis (model 3). In addition, Pearson's product-moment correlation was run to assess the relationship between ITNs and prevalence of Malaria in 259,885 participants.
