# Supplementary material for: Does livestock protect from malaria or facilitate malaria prevalence? A cross-sectional study in endemic rural areas of Indonesia
Source: Malar J. 2018 Aug 20;17:302. doi: 10.1186/s12936-018-2447-6 (PMC6102806; doi:10.1186/s12936-018-2447-6)
Supplement: Supplementary file 2 — Additional file 2. Detailed description of descriptive analysis and bivariate analysis. [file 12936_2018_2447_MOESM2_ESM.docx]

## Appendix S2: Detailed description of descriptive analysis and bivariate analysis.

## Descriptive analysis

*Characteristics of participants.* The proportion of females (50.7%) was slightly higher than the percentage of males (49.3%), while the proportion of participants who fell in the age range of 15-64 years was the greatest (60.1%). Furthermore, there were more participants, who had a low educated level (57.0%) than higher educated participants. Looking at employment, the proportion of participants whose main work were not a farmer, fisherman or labourer (45.4%) was higher than the other jobs category (31%) or category with participants <10 years [not worked] (23.6%).

*The accessibility & utilisation of health service.* The general access to health care was found to be good. The accessibility and utilisation of health services (facility: hospital, health centre, sub-health centre, Doctor’s practice, nurse practice) was good (93.2%) compared to poor access (6.8%). For primary health care, the access was also good (95.2%).

*Environmental sanitation.* Regarding environmental sanitation, participants who used a type of closed container (62.6%) was much greater than others (37.4%). However, the proportion of participants who had sewage canal from the bathroom/ laundromat/kitchen closed container (5.5%) was smaller than others and those who had a chemical sewage canal with the closed canal (9.9%).

*Behaviour of participants.* According to the behaviour of the participants, a greater proportion of them did not use mosquito nets (55.2%), and also, the majority of participants did not use insecticide-treated nets (29.0%) given that only 11.4% claimed to use a net insecticide (insecticide treated nets ITNs). The proportion of participants not using toilets was found to be slightly greater (55.7%) than those who did use toilets (44.3%).

## Bivariate analysis

*Characteristics of participants.* Malaria prevalence differed by gender. Males were more likely to have malaria than females (3.75% males *versus* 3.20% females, *P* < 0.001). Malaria prevalence also differed by age groups. The proportion of participants who in the age range of 15 - 64 years contracted malaria higher than others (3.67%; *versus* 3.17% , *P* < 0.001).

In addition, participants who had not completed high school education likelihood of contracting malaria than participants were considered as higher educated (3.52% *versus* 3.10%,, *P* < 0.05, but odds ratio = 1.00). Participants <10 years [pre-school] had a higher likelihood of contracting malaria than Participants were considered as higher educated (3.51% *versus* 3.10%, *P* < 0.05). The estimated odds of malaria from participants who had not completed high school education were 1.14 times higher than from participants who were considered as higher educated (OR = 1.14, *P* < 0.05).

Participants with a profession as a farmer/fisherman/labour have a higher likelihood of contracting malaria than participants who were not a farmer/fisherman/ labour (3.98% *versus* 3.23%, *P* < 0.001, but odds ratio = 1.00). Estimated odds of malaria for participants <10 years [not worked] was 1.24 times higher than for participants who were not a farmer/fisherman/labour (OR = 1.24, *P* < 0.001), although percentage for malaria is almost similar (3.25% *versus* 3.23%).

*The accessibility & utilisation of health service.* The proportion of participants, who stated that the accessibility and utilisation of health services at the hospital is good, contracted less malaria than those, who said that the access is not good (3.35% *versus* 5.03%, *P* < 0.001). Similarly, the proportion of participants, who reported that primary health care access is good, contracted less malaria than those, who said that the access was not good (3.37% *versus* 5.39%, *P* < 0.001).

*Environmental sanitation.* According to environmental sanitation, participants who did not possess of closed containers contracted malaria higher than participants who had others (3.68% *versus* 3.34%, *P* = 0.051). Similarly, participants who had not a closed chemical sewage canal from bathroom/laundromat/kitchen revealed a more likelihood of contracting malaria than others (3.51% *versus* 2.68%, *P* < 0.001). Also, participants who did not possess of a closed chemical sewage had a higher likelihood of contracting malaria than others (3.54% *versus* 2.85%, *P* < 0.001).

*Behaviour of participants.* The difference in malaria prevalence was only minor for participants who did not use mosquito nets with a slightly smaller likelihood of contracting malaria than participants who used mosquito nets at night (3.12% *versus* 3.84%, *P* < 0.001). However, odds ratio was 1.00 which implies that there is a similar risk of contracting malaria for two groups. Malaria in participants who did not answer was more likely prevalent than in participants who used mosquito nets at night (7.10% *versus* 3.84%, OR = 0.80, *P* < 0.001).

Participants who did not use insecticide-treated nets (ITNs) contracted malaria less likely than those who used mosquito ITNs (3.08% *versus* 5.89%, *P* < 0.001), but the risk for contracting malaria is equally distributed to two groups (OR = 1). Participants who did not answer contracted less likely malaria than those who used mosquito ITNs (3.19% *versus* 5.89%, OR = 0.51, *P* < 0.001). Indicating the limitation of bivariate analysis for the variable “use of ITNs”, there was a negative correlation between use of ITNs with the prevalence of malaria (r = 0.023, *P* < 0.001). This statistic implies the opposite to bivariate statistics: for participants who increasingly used ITNs, the prevalence of malaria decreased. Participants who did not use a toilet revealed not a significant increase of malaria prevalence in comparison to those who used a toilet (3.51% *versus* 3.41%, *P* = 0.051).

*The existence of livestock/pets*

Participants who kept the pets were more likely to contract malaria than those who did not keep pets (4.61% *versus* 3.08%, OR = 1.152, *P* < 0.001). Similarly, participants who raised poultry contracted more likely malaria than others (3.77% *versus* 3.11%, *P* < 0.001). Likewise, participants who raised medium sized breeding animals contracted more malaria than those who did not keep medium sized breeding animals (5.22% *versus* 2.97%, *P* < 0.001). On the contrary, keeping of large sized breeding animals did not significantly increase malaria prevalence (3.31% *versus* 3.49%, *P* = 0.43).

*Location of cages.* If untangling between indoor and outdoor caging, participants who kept caged pets outside the house were more likely to contract malaria than those keeping poultry indoors (5.32% *versus* 3.93%, *P* < 0.001). This difference in malaria prevalence was however only minor for participants who raised poultry either (4.19% outdoors *versus* 3.74% indoors, *P* < 0.001). Similiar the participants who kept medium sized breeding animals outside the house contracted malaria more than those who kept medium sized breeding animals indoors (8.35% *versus* 4.98%, *P* < 0.001). The participants who kept large sized breeding animals outside the house contracted malaria more than those who kept medium sized breeding animals indoors (4.15% *versus* 3.26%, *P* = 0.48).
